# Adaptive Multi-scale PHM for Robotic Assembly Processes

**Published:** 2015

**Authors:** Benjamin Y. Choo, Peter A. Beling, Amy E. LaViers, Jeremy A. Marvel, Brian A. Weiss

**Affiliations:** 1,2,3 University of Virginia, Charlottesville, Virginia, 22904, USA; 4,5 National Institute of Standards and Technology, Gaithersburg, Maryland, 20899, USA

## Abstract

Adaptive multiscale prognostics and health management (AM-PHM) is a methodology designed to support PHM in smart manufacturing systems. As a rule, PHM information is not used in high-level decision-making in manufacturing systems. AM-PHM leverages and integrates component-level PHM information with hierarchical relationships across the component, machine, work cell, and production line levels in a manufacturing system. The AM-PHM methodology enables the creation of actionable prognostic and diagnostic intelligence up and down the manufacturing process hierarchy. Decisions are made with the knowledge of the current and projected health state of the system at decision points along the nodes of the hierarchical structure. A description of the AM-PHM methodology with a simulated canonical robotic assembly process is presented.

## 1. Introduction

Prognostics and Health Management (PHM) refers to a class of techniques and methods that enable condition monitoring of a physical machine or functional process. PHM encompasses health monitoring of a system; provides diagnostic information including what is at fault, why the fault occurred, and how the fault can be remedied; and offers prognostic intelligence as to when a system or process is going to degrade to various states that may include going out of specification or failure.

A manufacturing system is a complex system-of-systems with a hierarchical structure. A manufacturing system hierarchical structure is described as a facility consisting of multiple assembly/fabrication lines that are further divided into work cells or work stations which are further divided into multiple machines consisting of components (Hopp & Spearman, 2008). One challenge in PHM for manufacturing is that in most applications data gathering and analysis is limited to the component level. For example, prognostic intelligence for machines, such as robots or machine tools, typically does not propagate beyond the boundaries of the machine even though the failure of a single component may lead to failure of other components or to system-wide effects.

The use of PHM technologies in manufacturing operations continues to experience growth driven by advances in sensor, computing, and communications technologies, and in machine learning and other data analytic techniques. An increased interest in PHM within manufacturing is also reflected in recent academic literature. Yoon, He, and Van Hecke (2014) applied PHM to an additive manufacturing process for improved fault diagnosis and quality control. Philippot, Marang, Gellot, Ptin, and Riera (2014) suggest a fault tolerant control structure for manufacturing plant control. The self-aware machine platform for application in a manufacturing shop floor proposed by Liao, Minhas, Rangarajan, Kurtoglu, and de Kleer (2014) provides a richer set of PHM information, including predicted component wear and real-time anomaly detection to the shop supervisor. However, there is a notable absence of methodologies to support the development of agile and flexible PHM systems in smart manufacturing environments (Peng, Dong, & Zuo, 2010).

Ideally, PHM would be available at the system level, including prognostic intelligence being propagated up the hierarchical structures that relate components to machines, machines to work cells, and work cells to production lines. Model-based diagnostic methods that have been developed for hierarchical aerospace systems may be applied to hierarchical manufacturing systems. For example, Narasimhan and Brownston (2007) suggested a general framework for stochastic and hybrid model-based diagnostics for aerospace systems. Feldman, de Castro and van Gemund (2013) proposed a decision support framework for satellite systems that uses active testing to increase diagnostic accuracy. Biswas and Mahadevan (2007) also proposed a framework for system health management that includes fault detection, fault identification, and adaptive control for aerospace applications. In the manufacturing domain, Celik, Lee, Vasudevan, and Son (2010) applied a dynamic data-driven framework on a supply chain system to perform multi-fidelity simulation. Ferri, Rodrigues, Gomes, de Medeiros, Galvo, and Nascimento (2013) have suggested a method for achieving system-level PHM by propagating the remaining useable life (RUL) along the fault tree structure of the manufacturing system model. This is a positive step in creating a methodology for achieving system-level PHM within Smart Manufacturing based on the system model and component-level PHM.

To address the existing gap in providing PHM for hierarchical manufacturing systems, we propose a methodology termed Adaptive Multiscale PHM (AM-PHM). The AM-PHM methodology is designed to support PHM in Smart Manufacturing Systems (SMS). AM-PHM is characterized by its incorporation of multi-level, hierarchical relationships and PHM information gathered from a manufacturing system. AM-PHM utilizes diagnostic and prognostic information regarding the current health of the system and constituent components, and propagates it up the hierarchical structure. By doing so, the AM-PHM methodology creates actionable prognostic and diagnostic intelligence along the manufacturing process hierarchy. This information includes the predicted health state upon completion of a task. The AM-PHM methodology allows for more intelligent decision-making to increase efficiency, performance, safety, reliability, and maintainability.

AM-PHM, at a given level along the system hierarchy, uses operational profiles from adjacent, higher-level operational profiles. These profiles describe the production goals under consideration by the decision-makers (e.g., operators and supervisors) at the higher level. In addition to the traditional workload, bill of materials, and requirements of the manufacturing process, the operational profile may have a focused objective such as minimizing cost or maximizing reliability. One instantiation of the AM-PHM concept may be as an AM-PHM module situated at every node along the hierarchical structure. The AM-PHM module gathers PHM information from subordinate systems or components and makes a decision ideal for the task corresponding to the operational profile. The AM-PHM module then creates operational profiles for its subordinate AM-PHM modules while producing diagnostic and prognostic information for its higher-level subsystem.

An example robotic assembly process is selected to show the effectiveness of the AM-PHM methodology. In today’s manufacturing world, the finished products/goods are becoming more complex as machines with increased capabilities are being deployed to the manufacturing floor. One example is the utilization of the industrial robot.

Worldwide, the manufacturing landscape has experienced extensive growth in the development and deployment of new robotic technologies. Paired with the introduction of newer, cheaper, and more reliable sensing technologies, the capabilities of robotic systems have improved in a relatively short amount of time. Processes that were historically performed by manual labor may now be accomplished using robots. As such, the use of robots outside of the automotive and electronics industries is on the rise (Orcutt, 2014).

Global manufacturing initiatives are stressing the development and integration of smart manufacturing technologies in modernized manufacturing facilities. Such technologies are seen as key to maintaining economic stability within an increasingly competitive global market (Holdren et al, 2011).

Robotic assembly is expected to be a principle application of robotics in manufacturing (Marvel & Falco, 2012). Historically, mechanical assembly has been addressed by manual labor. However, advancements in robotic perception, force control, and kinematic dexterity have enabled robotics to be viable options for assembly applications. This expands the traditional application suite of material handling, painting, and welding that have been more typical of robotic operations in manufacturing. Moreover, with the introduction of collaborative robot technologies, the expansion of robotics is expected to positively impact manufacturing processes that remain largely manual in nature (Marvel, 2014).

With the anticipated integration of robots into both new and preexisting manufacturing lines, the quality of PHM will directly influence the effectiveness of interoperability and system performance. This is particularly true when humans are expected to work alongside robotic collaborators, where robot performance also impacts safety. Should a robotic system experience a failure, it is expected to do so in a safe, reliable manner that does not negatively impact its environment, process, or collaborators. Moreover, the road to recovery must be clearly established and easy to implement. This necessitates significant advancements in the quality and dissemination of robotic PHM.

The remainder of the paper is organized as follows. Section 2 examines the current state of PHM capabilities and standards in manufacturing. Section 3 presents the AM-PHM methodology including the proposed AM-PHM features for describing the health state of systems. Section 4 discusses two example implementations of the AM-PHM methodology as applied to a test robotic assembly production line scenario. Section 5 concludes the paper by highlighting the significance of AM-PHM in manufacturing.

## 2. Current State of PHM in Smart Manufacturing

PHM technologies in manufacturing systems reduce time and costs for maintenance of products or processes through efficient and cost-effective diagnostic and prognostic activities. In 2010, a comprehensive review was conducted of prognostic and diagnostic methodologies for condition-based maintenance (CBM) that presented the existing strategies within four categories: physical models, knowledge-based models, data driven models, and combination (hybrid) models (Peng et al., 2010). This review highlighted many specific methods across four categories (Hidden Markov Models, Bayesian network-related methods, Fuzzy Logic, Principal Components Analysis) along with their successes and limitations. No method stood out as being sufficient to provide both diagnostic and prognostic intelligence at multiple levels. This review demonstrated that for every method’s strength, there was at least a single weakness. Similarly, another review of existing methods for manufacturing systems was conducted in 2012 that focused on comparing time-based maintenance (TBM) and condition-based maintenance (CBM) (Ahmad & Kamaruddin, 2012). TBM, commonly referred to as preventative maintenance, is typically simpler to implement (in that maintenance is scheduled based upon a specific unit of time; e.g., cycle time) while CBM, sometimes termed predictive maintenance, may ultimately be more cost effective if a process’s or equipment’s health data accurately reflects its current state and allows a machine to run longer until maintenance (as compared to a TBM schedule). The challenge in CBM is gathering sufficient data to make a reasonably accurate prediction.

Product PHM (providing health monitoring, diagnostics, and/or prognostics for a finished system; e.g., automobile, aircraft, power generation station) is more widespread as compared to process PHM (providing health monitoring, diagnostics, and/or prognostics to a system that integrates one or more pieces of equipment to complete a task; e.g., assembly process, welding process, machining process) (Batzel & Swanson, 2009) (Holland Barajas, Salman, & Zhang, 2010) (Hu & Koren, 1997) (Shen, Wan, Cui, & Song, 2010). Likewise, PHM techniques have been developed and applied more widely at component/equipment levels, yet some work has occurred at the higher/system levels. For example, innovative methods have been developed to support various machining operations (Al-Habaibeh & Gindy, 2000) (Altintas, Verl, Brecher, Uriarte, & Pritschow, 2011) (Biehl, Staufenbiel, Recknagel, Denkena, & Bertram, 2012) (Borisov, Fletcher, Longstaff, & Myers, 2013). System-level PHM methods have also been developed, yet seem to be focused in their applicability and/or limited in capability (Barajas & Srinivasa, 2008) (Datta, Jize, Maclise, & Goggin, 2004) (Hofmeister, Wagoner, & Goodman, 2013).

Vogl et al. (2014) conducted a detailed review of existing standards that were designed to help guide implementation of PHM in manufacturing. Specifically, many of the current PHM standards were developed within the International Organization for Standardization (ISO) and focus primarily on condition monitoring and diagnostics (ISO, 2002) (ISO, 2003) (ISO, 2012). Few standards include discussion of prognostics (ISO, 2004). Most standards fall into one of two categories; standards that are very specific and only applicable to a few processes and standards that are very broad that may lack guidance for applications. Likewise, no standard has been developed that offers the flexibility to be applied at multiple hierarchical levels of a complex system to promote effective PHM practices.

## 3. Adaptive Multiscale PHM for Smart Manufacturing

A manufacturing system hierarchical structure can be described as a facility consisting of multiple assembly/fabrication lines which are further divided into work cells or work stations which are further divided into multiple machines (Hopp & Spearman, 2008). For this paper, the hierarchical structure of the facility, assembly line, work cell, and machine will be used as a primary example, although there exists more complex methods of describing a manufacturing facility.

Information is passed down in the form of orders, schedules, bills of materials, or control signals between each hierarchical level of the system. The job of the subordinate system is to follow the tasks assigned by the higher-level node. Historically, maintenance policies for machines have been based on usage time or workload, as static policies defined in these terms can be estimated through historical data and experience. An effort to modify this approach into a feedback system where the health state of the machine or component is considered in making maintenance decisions emerged only recently. (National Institute of Standards and Technology, 2015) However, health state information is often confined to the component or machine level and is not propagated up to the system level.

On the other end of the spectrum, the system-level approach to analyzing a manufacturing system has resulted in generalized risk and fault analysis methods such as fault tree analysis (FTA) and failure mode and effects analysis (FMEA) (SAE International, 2009). Also, modeling software tools such as SysML have been used to describe the system structure including interoperability and interdependency between components of the system (Wünsch, Lüder, & Heinze, 2010).

**Figure 1:**
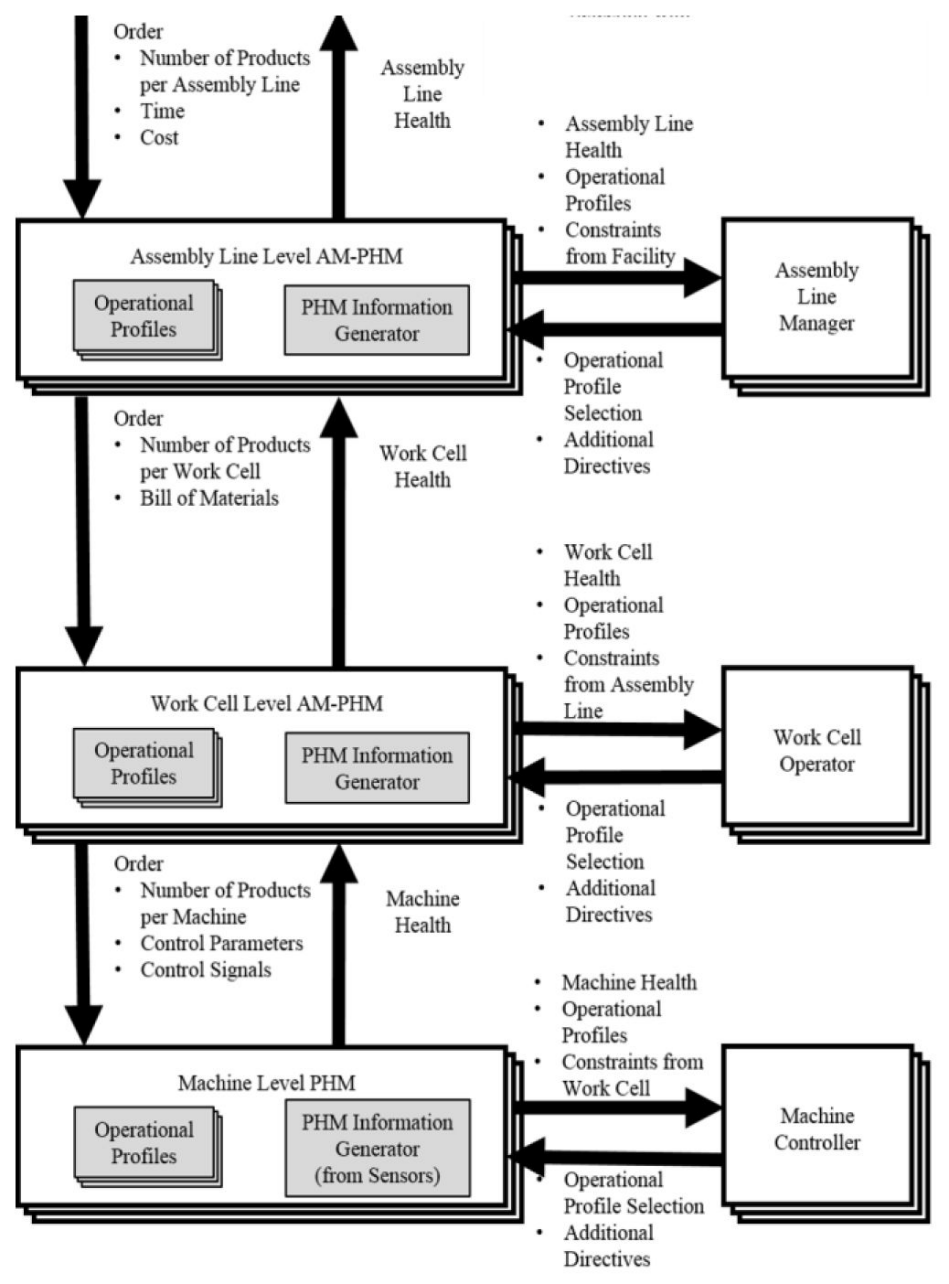
Conceptual representation of AM-PHM

The AM-PHM methodology is designed to provide decision-makers with enhanced information on the current and predicted health state of the decision-maker’s subsystems. Figure 1 depicts the AM-PHM methodology for a simple hierarchical manufacturing structure.

For AM-PHM, a decision-maker is not limited to the machine operator. Rather, it refers to any person or machine such as the control unit of a manufacturing robot or the supervisor of an assembly line that is responsible for making decisions that can influence the outcome of the system. The point at which the decision-maker resides in the hierarchical structure is called the decision point within the AM-PHM methodology. Conceptually, an AM-PHM module resides at every decision point of the hierarchical structure of the manufacturing system.

A hierarchical manufacturing system refers to a manufacturing system in which multiple levels exist. For each level, the higher-level nodes encompass the lower-level nodes. In this level structure, the parent nodes have control over the states of its subsystems while subsystems do not have direct control over the states of its parent nodes. Examples of the hierarchical structure may be a SysML description or a fault tree structure of the manufacturing system. Another example may be a treelike description of the physical setup of a manufacturing system consisting of assembly lines, work cells, and machines.

For the example structure shown in Figure 1, an order is placed to the Facility Manager with the number of products requested, product requirements, and expected finish date. The order information and the operational directive are passed onto the facility level AM-PHM module. The directive refers to a particular set of attributes or objectives that the decision-maker would like to focus on. For example, the decision-maker may be interested in reducing the time, cost, risk, or wear in maximizing the utilization rate.

The facility level AM-PHM module reports the health information of the facility to the Facility Manager. PHM information on the subsystem is needed for effective directive-driven decisions to be made. The PHM information from the subsystem is processed at each AM-PHM module. This results in health metrics that appropriately represent the current and future state of the system. These health metrics may include remaining usable life of the system, expected health state upon completion, nature of fault, and proposed solutions.

The AM-PHM module also creates operational profiles once all aforementioned information is gathered. Each operational profile is designed to control the subsystems with a focused directive. The operational profile also contains the projected health information for the subordinate systems such as projected health upon completion. The decision-maker may now choose from the set of operational profiles that fit within the constraints handed down from its superior nodes.

The Facility Manager selects the operational profile that best fits the directive and order requirements. Once the operational profile is chosen, the set of instructions contained within that operational profile are handed down to the subordinate AM-PHM module and a similar process repeats itself. For the example in Figure 1, the selected operational profile containing the number of products needed to be produced by each production line and operational directive is passed down to the Assembly Line level.

A similar process is now repeated at the Assembly Line level. The Assembly Line Manager takes the operational profile handed down from the Facility level and selects an appropriate operational profile. The operational profile handed down to the Work Cell level contains information such as number of products produced for a particular work cell and bill of materials needed for the processing of the order.

A similar process is repeated for the Machine level. For the Machine level the operational profile contains machine operation parameters and the AM-PHM information contain data such as the aggregated wear for critical components.

Although the simplified scenario depicted in Figure 1 is convenient for initial discussion of the AM-PHM concept, the concept may be expanded for more general SMS environments in which there exists an extensive hierarchy of processes and components.

Additional features that better describe the current health state at a particular juncture of the system are needed for the AM-PHM system to be helpful to the decision-maker. The newly suggested features are (a) *greatest wear*, (b) *average wear*, (c) *health balance score*, (d) *probability of successful completion*, and (e) *estimated health state upon completion*.

- *Greatest wear* represents the most extreme wear in percent from all the wear states of all the subordinate components. This gives an idea of the state of the most worn component of the system.
- *Average wear* represents the arithmetic weighted mean of the wear in percentage of all the subordinate components. This metric represents the overall average health state of the system. The average on its own may not reveal much information but in conjunction with the *greatest wear* and the *health balance score*, this helps to describe the health state of the all the components of the subsystem. Different components contribute differently to the overall performance of a manufacturing system. There are established methods such as FTA, FMEA, Hierarchical Holographic Modeling (HHM), and Risk Filtering, Ranking, and Management (RFRM) that may be used to analyze the weight of each component to different failures. The differing importance of a component is included as a weighted coefficient.
- *Health balance score* is the standard deviation of the wear state of each of the subordinate components at a given node. This metric indicates degree of concentration of wear of the system. A higher number would indicate that wear values vary greatly among components, while a smaller number would indicate that the system has similar wear along most of its components.
- *Probability of successful completion* is the probability that the component will complete the given operational profile with the current state of health. This gives decision-makers an idea of the success rate or confidence involved with a given solution.
- *Estimated health state upon completion* refers to the expected final state of health for all metrics involved in AM-PHM. This is used to show a predicted picture of the overall state of health at the point of completion of the assigned task.

The *average wear*, *health balance score*, and *probability of successful completion* may be further customized so that each of the components carry different weights. This means the proposed metrics can focus on certain components depending on its importance within the overall structure of the system.

One notable point for the proposed features is that the basis for the usefulness of these metrics lies on the assumption that the PHM information from the component level is accurate to a certain degree. An accurate wear model is necessary for the health metrics to be useful.

## 4. AM-PHM Implementation in Smart Manufacturing Robotic Assembly

An example assembly line involving multiple robots is described in this section. The AM-PHM methodology is applied to the canonical manufacturing process simulation. The canonical process is a generalized test case of the example assembly line and includes related assumptions. This simplified test case, including its simulated results, highlights the usefulness of the AM-PHM implementation. The structure and the trend of the numbers involved are reasonable in real manufacturing settings. The use of robotic arms in industry is widespread as stated in Snyder (1985) and the trend of the drill wear in the canonical example simulation follow the model by Kadirgama, Abou-El-Hossein, Noor, Sharma and Mohammad (2011).

The example hierarchical structure of a manufacturing environment consists of a single assembly line with multiple work cells, each of which has multiple machines, each comprised of multiple components. The operational profiles flow from the higher-level block to the lower-level blocks in the AM-PHM framework. The PHM information is reported from the lower-level blocks up to the higher-level block. However, both the operational profile and the PHM information are processed appropriately for each level.

The specific information that is listed in the operational profiles and the PHM reports differ depending on the block’s location in the hierarchical structure. For example the operational profile generated by the assembly line for each work cell will resemble a bill of materials; the operational profile generated by the work cell for each machine will resemble a process instruction; and the operational profile generated by the machine to its components will be close to a set of control signals.

The operational profile generator of the AM-PHM module at each level must translate the task it receives from the higher-level AM-PHM module into a task that can be understood by the subordinate level. Similar concepts apply to the PHM information at each stage. The PHM information from the component to the machine will include RUL of replaceable parts, while the PHM information from the machine to the work cell includes more information on the tradeoffs involved with different operational profiles. Finally, the PHM report from the work cell to the assembly line would include more information on the probability of successful completion and the overall health state of the work cell. The AM-PHM module must process the PHM information it receives from the lower levels and provide value-added, level appropriate information for the upper level.

Two different examples of AM-PHM are given. The first example is focused on a simple AM-PHM structure with simple operational profiles and a PHM report involving only RUL. This structure may be implemented if the nature of the task performed at an assembly line does not require sophisticated PHM capabilities or if changing the existing system model and fault tree structure is not desired. The deployment of AM-PHM into the existing assembly line model is minimally invasive and most likely will not affect the overall structure of the fault tree.

The second example is a more sophisticated AM-PHM system. This is needed if the assembly line handles a more complex process involving many different machines with interdependencies and interoperability. The downside is that the implementation may become more complicated and the use of AM-PHM may impact the existing fault tree structure of the system model.

**Figure 2:**
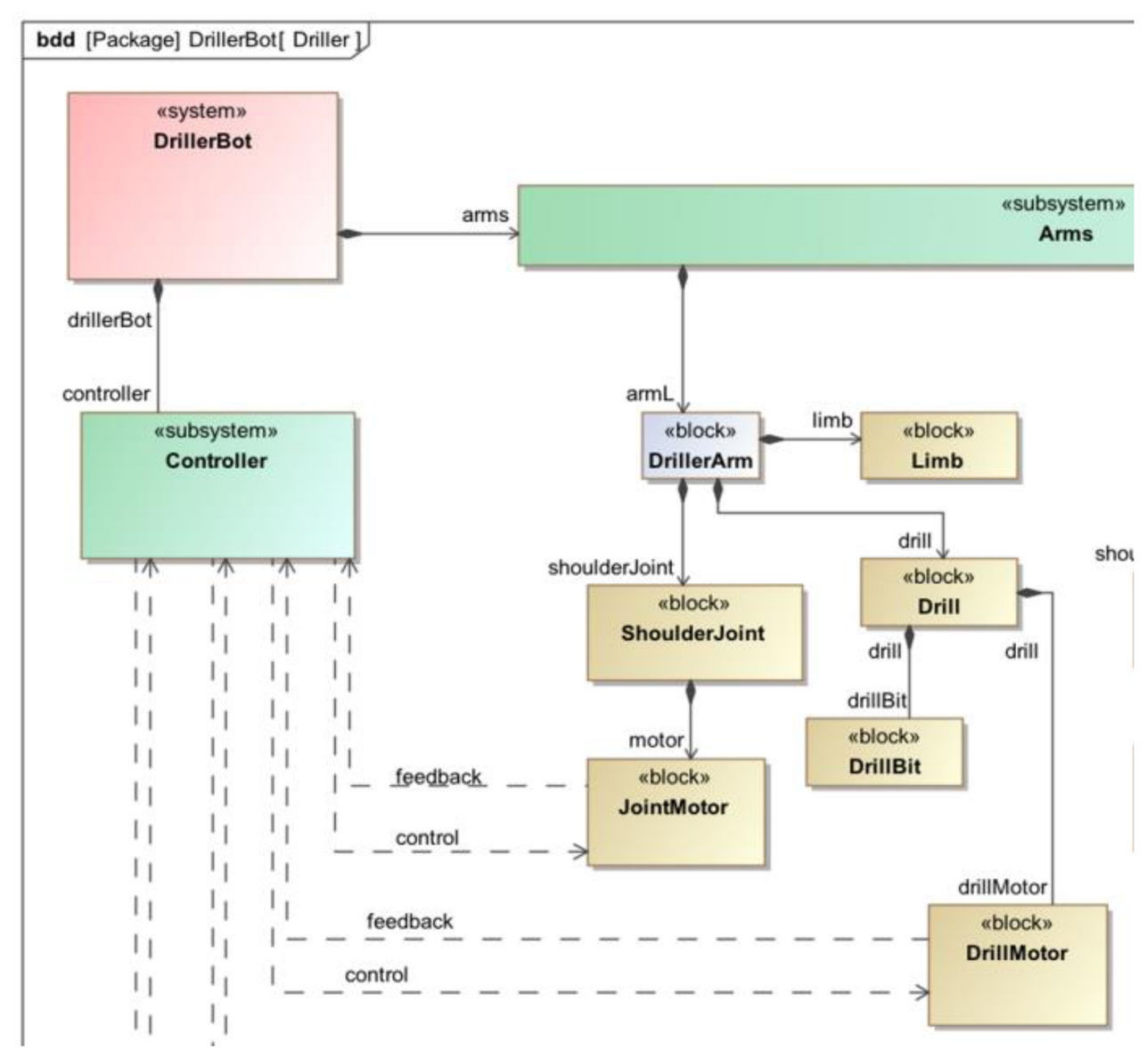
SysML description of drilling robot

**Figure 3:**
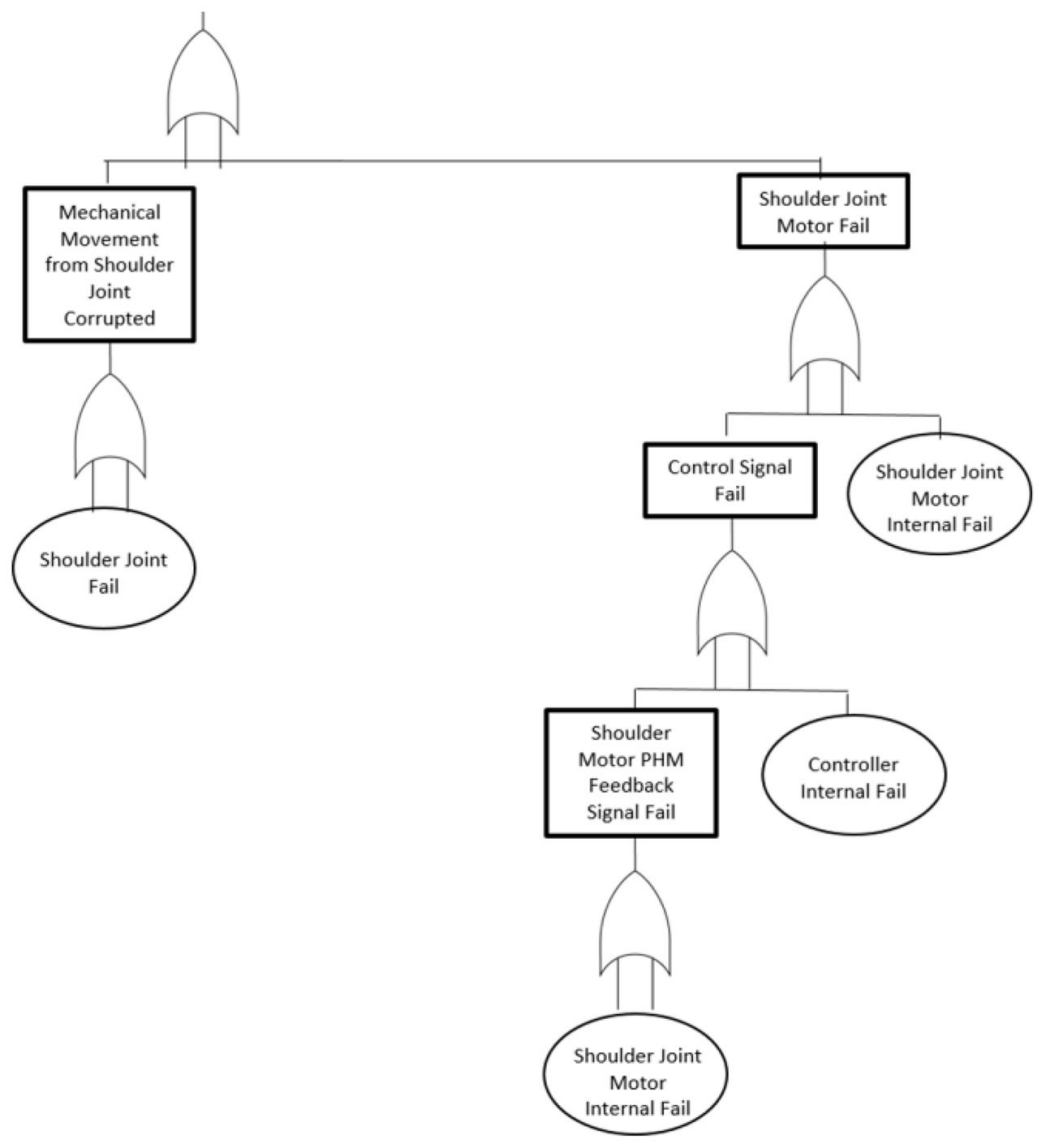
Fault tree analysis (FTA) of drilling robot

In the canonical example, a robot with two drilling arms is used to drill holes into a box. The left and right drilling arm are each responsible for drilling holes into the left and right side of the box, respectively. A SysML model of the drilling robot is presented in Figure 2. The corresponding FTA diagram of the drilling robot is shown in Figure 3. Only the flank wear of the drill bit component on each arm is considered for the simplified AM-PHM example, as flank wear is one of the common wears exhibited in drilling (Kadirgama et al. 2011). It is important to note that one drilling arm may perform the job of the other drilling arm with the penalty of reduced production rate.

In real-world manufacturing systems, there are many factors such as material properties, work piece structure, and machine characteristics that are carefully considered when selecting machining parameters. Machining parameters are optimized to best fit the particular manufacturing process. However, in a complex system-of-systems, optimization based on one feature means there is a trade-off with other features. Also, for a particular process there is a range of acceptable machining parameters rather than one fixed operating point (Furness, Wu, and Ulsoy, 1996). Drill bit manufacturers recommend a range of feed rates and cutting speeds for their drill bits (Sandvik Coromant, 2005).

When the parameters for a process are selected, the model [for the process] does not account for the fact that the system may change as the machine experiences wear in its components. The wear of the components, such as the flank wear of the drill bit, affects the characteristics of the system. Thus, the optimal operating parameters may need adjustment to account for the change in the system caused by the deteriorating health state of the machine.

For the canonical process example simulation, simplifications are made to emphasize the effect of the AM-PHM methodology and to reduce the complexity of the example. The drilling robot is tasked to drill 100 holes on the left and right side of the box. The left and right drilling arm each drill on their respective sides, simultaneously. Though there are several different types of wear involved with the drill bit, only the flank wear occurring on the cutting edge of the drill bit is considered.

The work piece is made of Nickel alloy with a Brinell hardness of 200. The production line has identified an acceptable and stable range of operating parameters. The cutting speed is between 100 m/min to 180 m/min. The feed rate is between 0.1 mm/rot and 0.2 mm/rot. Each hole has a cutting depth of 1.5 mm and the drill diameter is 10 mm. Expected tool life is different for different combinations of cutting speed and feed rate and follows the values stated by Kadirgama et al. (2011).

The drill bit is considered completely worn and reached its replacement point when there is 0.3 mm of flank wear. The RUL or tool life depends on the machining parameters and the replacement threshold for the drill bit. Tool life also differs depending on the size and geometry of the drill bit. Thus, to provide a more comparable quantitative figure for the amount of wear, the wear is presented as a percentage. The wear percentage is calculated by dividing the remaining tool life by the tool life for a new tool.

### 4.1. Simple Implementation of AM-PHM

The AM-PHM module is implemented on the canonical example robotic assembly process. Only the RUL is propagated based on the system’s SysML model to provide RUL information along the system’s hierarchical structure.

Two methods by Mhenni et al. (2014) and Ferri et al. (2013) were combined to achieve this task. Mhenni et al. (2014) suggested a method for converting a SysML model into a fault tree. The method uses templates that translate several basic SysML subcomponent blocks into an equivalent fault tree structure. Then rules are suggested for combining these small fault trees into a complete system fault tree. Figure 3 shows the fault tree constructed using this automated algorithm. The leaf nodes of the fault tree correspond to the individual components of the left arm of the driller robot.

Another example PHM technique that could be applied within AM-PHM was developed by Ferri et al. (2013). This research team developed a method for propagating the RUL along a fault tree. This methodology takes the RUL of the end components and applies a set of rules to produce the RUL at each node of the fault tree. The PHM capability provides the RUL for each component. The individual component-level RUL is combined, resulting in the overall RUL for the driller robot.

A semi-automated method for building system-level AM-PHM is completed through the combination of these two methods. The system-level RUL is produced given the availability of the SysML model and component-level RUL. In this case, the actual implementation of the AM-PHM is done through the use of FTA as an intermediate, semiautomated step of linking system-level hierarchical information and component-level health information. Only the RUL given at each level is used as the source of health information.

The work cell is tasked to build 20 boxes. The starting wear state of the individual drill bits are 85 % worn for the left drill in Robot 1 and new for all other drill arms. The default operating speed is set to a feed rate of 0.2 mm/rot and 120 m/min. This results in a wear rate of 15 % per minute for the drill bit and a production rate of 5 boxes per minute. The component-level RUL is calculated based on this initial condition. The component-level RULs show that for Robot 1, the left arm has an RUL of 1 minute and the right arm has an RUL of 6.6 min. For Robot 2, both the left and right arm has an RUL of 6.6 min. This information is propagated along the hierarchical structure according to the rules. Robots 1 and 2 each result in RULs of 1 minute and 6.6 min, respectively. The decision to distribute the load to the two robots is made based on the production targets and RUL information by the work cell operator. A work load of five boxes is assigned to Robot 1 and a work load of 15 boxes is assigned to Robot 2. The job takes three min to complete and the final RUL upon completion for each robot is 0 and 3.6 min, respectively. The complete results including additional information on the health of the work cell are presented in Table 1.

The result shows that the system has an RUL of 3.6 min. This is information previously unattainable to the decision-maker. Utilization of the RUL information enables more efficient use of the components of the manufacturing system. The advantage of this degree of PHM reflection is that at any point in the hierarchical structure the same RUL calculation method can be applied again reducing the complexity of implementation. The upper-level RUL is calculated using simple multiplication and comparison process. This begins by converting the fault tree (consisting of logic AND and OR gates) to a sum of products (SOP) expression. Once the SOP expression for the system is obtained, the system RUL is calculated by multiplying the probability distribution of the RULs for the product terms of the expression. The next step is to select the appropriate RUL for the sum portion of the expression. The system RUL ends up highlighting the set of components that are contributing to the nearest expected system failure. However, the system RUL does not contain health information on the other components of the system that are not directly tied to the upcoming failure. This limits the range of intelligent decisions that can be made.

### 4.2. Full Implementation of AM-PHM

A more sophisticated implementation of the AM-PHM concept would be to introduce additional features that help convey timely information on the health state of the system. The new features used in this example are the *health balance score*, *average wear state*, *worst wear state*, and *estimated wear state upon completion*. An order to make five boxes was given to the work cell as with the previous example. For the starting health state, only the right drill arm’s wear state is at 75 % while all other components are new.

The PHM information from a subordinate component is conveyed to the upper-level AM-PHM module. The collected PHM information is processed to produce the PHM information at the current node. The cutting speed and feed rate parameters are changed to a different operating point within the stable and acceptable range. Work load is changed and the expected results are calculated for all the different parameters. The drill bit wear trend follows the model suggested by Kadirgama et al. (2011). The production rate is changed by adjusting the cutting speed and feed rate which effects the wear rate of the drill bit. According to Furness, Wu and Ulsoy (1996) the feed and speed have relatively small effects on the drill hole quality and that the drilling feed and speed is limited by factors such as drill wear. The drill speed parameters may be adjusted within a certain confine without significantly affecting the hole quality. The final decision is made from the set of choices that best fits the operational directive. The results for this simulation are given in Tables 2 and 3.

For the case in Table 2, the work cell was handed down orders to produce 20 boxes with a directive of minimum health balance. Low balance score means that the components are at a similar state of health and may be used to align maintenance points for the components. The chosen operational profile distributes a load of five boxes for the first robot and 15 boxes for the second robot. However, the cutting speed is adjusted to 100 m/min and the feed rate is also adjusted to 0.1 mm/rot. The production rate is slowed down to 2.1 box/min as a result which reduces the wear of Robot 1’s drill bits to 0.02 mm/min or 6.6 % of its tool life per minute. This results in the production taking approximately 2.1 min.

For the case in Table 3, the work cell is also ordered to produce 20 boxes but with a directive of minimum time. The operational profile chosen suggests a cutting speed of 180 m/min and a feed rate of 0.2 mm/rot. The production rate is increased to 7.6 boxes per minute at the cost of seeing 0.1 mm of flank wear per minute or 33 % of reduction in tool life per minute. The left drill bit reaches its failing point after 30 seconds and the right drilling arm handles the job of drilling holes on the left side as well which reduces the production rate for Robot 1. Production is completed in 1.5 min at an increased cost on the wear of the drill bits.

The AM-PHM methodology is being applied in a simulated environment that is designed to resemble real-world hierarchical manufacturing systems. The canonical example simulation is based on real-world drill bit wear trends. For simplicity, in this paper, tool life is only dependent upon the operating parameters since the material stays consistent. The AM-PHM suggests operating points by optimizing a weighted cost function. The cost function includes all the health related features. The weight used in the cost function is adjusted depending on the decision-maker’s operational directive. The suggested actions such as changes in parameters are based on existing stable operating conditions to ensure system stability.

The canonical simulation used in this example is based on models from literature. In the future the AM-PHM methodology will be applied to real-world data some of which is obtained from actual production facilities. The real data will also include a more detailed wear model in which the wear rate is also dependent on additional factors such as current state of wear and material properties.

## 5. Conclusion

The concept of Adaptive Multiscale PHM for manufacturing was introduced in this paper. The AM-PHM methodology calls for the AM-PHM module at each decision point along the hierarchical structure to receive operational profiles outlining the job requirements and report back performance and health estimates appropriate for the upper level.

The AM-PHM is demonstrated on a canonical test manufacturing scenario simulation. Directive oriented decisions were made in the simulation by using additional information on the health of the system in addition to knowledge on the system hierarchical model. The AM-PHM shows promising results as it enables manufacturing work cells to adapt to changing machine conditions.

Further development of the AM-PHM methodology will continue. A modified work cell canonical process is in development. This model is based on a real-world manufacturing facility. A canonical process work cell simulator capable of simulating continuous wear of the components is being developed. The AM-PHM will be tested using this simulation environment and will be compared against other existing PHM based decision-making policies. The results of the different policies will be compared using quantitative measures such as time, monetary cost and Overall Equipment Effectiveness (OEE).

## Figures and Tables

**Table 1:** AM-PHM based manufacturing results using RUL

Time (min)	0	1	2	3
Production Rate of Robot 1 (box/min)	5	5	-	-
RUL of Robot 1 (min)	1	0	-	-
Production Rate of Robot 2 (box/min)	5	5	5	5
RUL of Robot 2 (min)	6.6	5.6	4.6	3.6
Produced (box)	0	10	15	20
RUL of Work Cell 1 (min)	6.6	5.6	4.6	3.6

**Table 2:** AM-PHM results based on maximum mean health

Time (min)	0	1	2	3
Production Rate of Robot 1 (box/min)	2.1	2.1	2.1	-
RUL of Robot 1 (min)	15.15	14.15	13.15	13
Production Rate of Robot 2 (box/min)	7.6	7.6	7.6	-
RUL of Robot 2 (min)	3	2	1	1
Produced (box)	0	9.7	19.4	20
RUL of Work Cell 1 (min)	15.15	14.15	13.15	13.15

**Table 3:** AM-PHM result using maximum health balance and minimum time

Time (min)	0	1	2	3
Production Rate of Robot 1 (box/min)	7.6	5.7	3.8	0
RUL of Robot 1 (min)	3	2	1	1
Production Rate of Robot 2 (box/min)	7.6	7.6	7.6	0
RUL of Robot 2 (min)	3	2	1	1
Produced (box)	0	15.2	20	20
RUL of Work Cell 1 (min)	3	2	1	1
